# Are health care professionals able to judge cancer patients' health care preferences correctly? A cross-sectional study

**DOI:** 10.1186/1472-6963-10-198

**Published:** 2010-07-08

**Authors:** Hester Wessels, Alexander de Graeff, Klaske Wynia, Miriam de Heus, Cas LJJ Kruitwagen, Saskia CCM Teunissen, Emile E Voest

**Affiliations:** 1 Department of Corporate Communications, University Medical Center Utrecht, Heidelberglaan 100, 3508 GA, Utrecht, The Netherlands; 2 Department of Medical Oncology, University Medical Center Utrecht, Heidelberglaan 100, 3508 GA, Utrecht, The Netherlands; 3 Graduate School for Health Research (SHARE), University Medical Center Groningen, Hanzeplein 1, 9700 RB, Groningen, The Netherlands; 4 Julius Center for Health Sciences and Primary Care, University Medical Center Utrecht, Heidelberglaan 100, 3508 GA, Utrecht, The Netherlands

## Abstract

**Background:**

Health care for cancer patients is primarily shaped by health care professionals. This raises the question to what extent health care professionals are aware of patients' preferences, needs and values.

The aim of this study was to explore to what extent there is concordance between patients' preferences in cancer care and patients' preferences as estimated by health care professionals. We also examined whether there were gender differences between health care professionals with regard to the degree in which they can estimate patients' preferences correctly.

**Methods:**

To obtain unbiased insight into the specific preferences of cancer patients, we developed the 'Cancer patients' health care preferences' questionnaire'. With this questionnaire we assessed a large sample of cancer patients (n = 386). Next, we asked health care professionals (medical oncologists, nurses and policymakers, n = 60) to fill out this questionnaire and to indicate preferences they thought cancer patients would have. Mean scores between groups were compared using Mann-Whitney tests. Effect sizes (ESs) were calculated for statistically significant differences.

**Results:**

We found significant differences (ESs 0.31 to 0.90) between patients and professionals for eight out of twenty-one scales and two out of eight single items. Patients valued care aspects related to expertise and attitude of health care providers and accessibility of services as more important than the professionals thought they would do. Health care professionals overestimated the value that patients set on particularly organisational and environmental aspects.

We found significant gender-related differences between the professionals (ESs 0.69 to 1.39 ) for eight out of twenty-one scales and two out of eight single items. When there were significant differences between male and female healthcare professionals in their estimation of patients health care preferences, female health care professionals invariably had higher scores. Generally, female health care professionals did not estimate patients' preferences and needs better than their male colleagues.

**Conclusions:**

Health care professionals are reasonably well able to make a correct estimation of patients preferences, but they should be aware of their own bias and use additional resources to gain a better understanding of patients' specific preferences for each patient is different and ultimately the care needs and preferences will also be unique to the person.

## Background

Health care providers search for solutions to deliver patient centered care that is respectful of and responsive to individual patient preferences, needs and values. Although patient advocacy groups have increasing influence on health care organizations, health care is mostly shaped by health care professionals. An important condition for health care professionals to provide optimal patient centered care, is a good insight into the patients' needs and preferences concerning health care [1,2].

To obtain insight in the specific preferences of cancer patients, we have developed the 'Cancer patients' health care preferences' questionnaire', based on patients' unrestricted input [3]. This questionnaire is not a satisfaction questionnaire, but a questionnaire that evaluates the importance of care aspects. The questionnaire was given to a large sample of patients treated by medical oncologists. In a previous study [3], it appeared that patients set highest value on safety and on the expertise, attitude and communicative skills of doctors and nurses. Of relatively less importance are the organisational and environmental factors.

Patients with cancer may encounter physical, existential and emotional problems. Information about cancer patients' needs and preferences can, to some extent, be derived from the literature [4-14]. Still, tailoring care as much as possible to the patients' needs and preferences will especially be based on the insight of the individual health care professional. This raises the question to what degree health care professionals are able to estimate patients' needs and preferences correctly.

Previous research examining the estimation of patient needs by health care professionals focuses on the impact of cancer and the side effects of chemotherapy [15], psychological concerns and needs [16,17] and patient reported physical and psychosocial problems [18,19]. These studies show that there is a considerable discrepancy on various issues between patients' and health care professionals' perceptions. Lack of concordance between patients and health care providers may result in less than optimal health care. In relation to these findings Brennan et al. [20] state that if nurses, physicians and health care planners knew more about patients' health-related preferences, care would most likely be cheaper, more effective and closer to the individuals' desires.

To our knowledge, the question of whether health care professionals estimate cancer patients' preferences for health care (in general) correctly, has not been studied systematically. Therefore, the aim of the present study was to examine to what extent there is concordance between cancer patients' preferences for health care, and the estimation of patient preferences by health care professionals.

In a previous study we found significant differences between male and female patients concerning their preferences in health care [21]. As gender differences with regard to communication style, practice style and relationship with patients were also found for health care professionals [22-25] we decided to examine additionally whether there are gender differences between health care professionals with regard to the degree in which they can estimate patients' preferences correctly.

## Methods

### Questionnaire and patients

The research protocol was approved by the Medical Ethics Commission of the University Medical Center Utrecht.

The development of the 'Cancer patients' health care preferences' questionnaire' has been described elsewhere [3]. Briefly, items concerning preferences for health care were generated during focus group interviews with cancer patients. Patients for the focus group interviews were recruited by medical oncologists of the University Medical Center Utrecht and through the Dutch Federation of Cancer Patient Organisations. Eligible patients had a sufficient physical condition to participate in the interview and were able to speak and understand the Dutch language. Finally 51 patients participated. Patient characteristics were: 67% female, 39% <35 year, main tumour types were breast (18%), urological (20%), genital (10%) and gastrointestinal (6%). During the two hours lasting focus groups only one question was put forward by the panel leader: 'How would you design health care if you were in charge?' Participants were explicitly asked to think out of the box and forget potential constraints. Each interview was digitally recorded and transcribed. Text fragments were independently coded and categorized by two authors (HW, MdH). Based on analysis diagrams questions were formulated by two researchers and a questionnaire containing 136 items was generated. Each item evaluates the level of importance on a 4-point scale, ranging from 'Not important' (1), 'Somewhat important' (2), 'Important' (3), to 'Extremely important' (4).

The questionnaire was distributed among patients in care of medical oncologists from one university medical center and six community hospitals in the region of Utrecht, the Netherlands. Doctors and nurses of these departments handed out the questionnaires to an unselected sample of consecutive cancer patients. The questionnaires were encoded by hospital. A cover letter informed patients about the aim of the study and the importance of their input. Respondents were assured that their answers would be kept confidential and that the data would be processed anonymously. A phone number and email-address to contact the project manager were provided. Respondents could complete the questionnaire at home and send it back anonymously in a self addressed pre-stamped envelope. A reminder was sent to each patient after four weeks. The Medical Research Ethics Committee judged that it was not necessary for patients to sign a consent form for the study.

An explorative factor analysis with Varimax rotation was performed, resulting in the final questionnaire containing 21 scales with 115 items and 8 single items. Internal consistency of the scales was sufficient to good. The process of deleting and including items into scales is described elsewhere [3].

All scores of scales and single items are transformed to a score of 0 -100, with high values indicating a high level of importance.

### Health care professionals

Health care professionals involved in the delivery and organisation of care for cancer patients (doctors and nurses of the departments of medical oncology and members of hospital management) of the seven participating hospitals were asked to participate in the study and to fill out the 'Cancer patients' health care preferences' questionnaire' [3]. Per participating hospital questionnaires were handed to a contact person who distributed the questionnaires to the health care professionals. We asked respondents to indicate health care preferences they thought cancer patients would have. Questions regarding respondents' gender, age and discipline were added. A cover letter informed participants about the aim of the study and the importance of their input. Respondents returned the questionnaire anonymously. A reminder was sent to each health care professional after three weeks.

### Data analyses

Data were analysed using SPSS version 15.0 (SPSS Inc., Chicago, IL). Mean scores between groups were compared using Mann-Whitney tests. In case of significant differences (p < .05) between groups, effect sizes (ESs) were calculated to estimate the magnitude of these differences. According to Cohen's thresholds [26], an effect size of <0.20 indicates a trivial effect, an ES of ≥0.20 to <0.50 a small effect, an ES of ≥0.50 to <0.80 a moderate effect and an ES of ≥0.80 a large effect. An ES ≥0.20 reflects a relevant difference between groups [27].

## Results

### Patients and health care professionals

Between October 2006 and March 2007, 681 questionnaires were handed out to patients. In total 386 questionnaires were returned (57% response rate) and included in the analysis. Characteristics (based on self-report) of these patients are summarized in Table 1.

**Table 1:** Characteristics of patients and health care professionals

Characteristic	Patients	Health care professionals
	(n = 386)	(n = 60)
	**Percent**	**Percent**
**Hospital**		
University Medical Center	27	35
Affiliated hospital	73	65
**Sex**		
Male	35	20
Female	66	78
Unknown	-	2
**Age, years**		
18-35 years	5	32
36-50 years	28	53
51-65 years	38	12
66-79 years	26	-
Unknown	4	2
**Level of education**		NA
Less than high school	9	.
High school	62	.
More than high school	30	.
**Discipline**	NR	
Physician	.	13
Nurse	.	67
Policymaker	.	10
Unknown	.	10
**Type of cancer patients were treated for**		NR
Gastrointestinal	21	.
Breast	45	.
Skin	1	.
Urological	10	.
Genital	10	.
Head and neck	2	.
Lung	1	.
Other	12	.
**Type of treatment **(concurrent or previous)*		NR
Chemotherapy	78	.
Hormonal therapy	26	.
Experimental treatment	4	.
Radiation therapy	46	.
Chemo radiation	3	.
Surgery	72	.
Other	NA	.
**Stage**		NR
Metastases present	72	.
Metastases absent	28	.

* Patients could tick off several answers

NA = not applicable/not asked; NR = not relevant

Between May and August 2007, 165 questionnaires were distributed to health care professionals. Sixty questionnaires were returned (36% response rate) and included in the analysis. Characteristics of the health care professionals are provided in Table 1.

### Comparison of results of patients and health care professionals

Table 2 shows the results of the scales and single items (ranked in level of importance according to the mean scores as indicated by the patients) for both health care professionals and patients. Overall there is a strong correlation between both groups (Spearman correlation coefficient 0.92).

**Table 2:** Comparison of the importance scores of health care professionals and patients

	Health care professionals (n = 60) mean (SD)	Patients (n = 386) mean (SD)	Profs vs Patients ES#
**Scales**			

Mistakes by professionals	86 (15)	90^1 ^(13)	
Physician and nurse expertise	85 (13)	89 (11)*	- 0.35
Consultation and transfer	83 (15)	84 (14)	
Physician attitude	76 (13)	81 (13)**	- 0.38
Patient file confidentiality	85 (16)	81 (18)	
Opportunity to choose in care and treatment	78 (13)	80 (14)	
Nurse attitude	80 (16)	78 (14)	
Communication and information	78 (10)	77 (12)	
Accessibility of services	72 (13)	77 (14)**	- 0.36
Waiting periods	76 (13)	76 (16)	
Support, counseling and rehabilitation	67 (15)	61 (20)*	+ 0.31
Alternate sources of information	64 (13)	60 (23)	
Appointments	62 (14)	59 (18)	
Rooms and facilities	55 (14)	57 (14)	
Food and beverages	56 (22)	56 (19)	
Presence of loved ones	57 (21)	49 (26)	
Privacy	54 (18)	46 (22)*	+ 0.37
Patient habits	50 (18)	43 (22)**	+ 0.33
Patient interest groups	45 (20)	37 (23)**	+ 0.35
Conveniences	44 (16)	37 (16)**	+ 0.44
Fellow-patient interaction	19 (16)	17 (19)	
**Single items**			
Hospital equipment	66 (20)	84 (20)***	- 0.90
Consultation at ER by own doctor	70 (23)	79 (20)**	- 0.44
Written information	78 (20)	77 (21)	
Support of a case manager	69 (22)	74 (24)	
Continuity in care	67 (20)	72 (22)	
Support by paramedical staff	68 (16)	68 (18)	
Attention for nutrition	63 (23)	68 (22)	
Leaving choices to doctors and nurses	62 (28)	66 (32)	

^1^A higher score indicates a higher level of importance (range 0-100);

*p < .05, **p < .01, ***p < .001 (Mann Whitney tests)

# Effect size (ES) only calculated for scales and items tested as statistically significant different.

+ ES: the professionals score is higher than the patient score; - ES: the professionals score is lower than the patient score.

However, for 8 of the 21 scales and 2 of the 8 single items, we found statistically significant differences between health care professionals and patients. All effect sizes of these scales and single items were between 0.31 and 0.44 with the exception of the single item concerning the quality of hospital equipment, which had a very strong effect size (0.90).

Of these ten scales and single items, patients rated five scales and single items as more important than health care professionals expected: 'Physician and nurse expertise' (items concerning knowledge and experience, complete information about the patients situation and specialisation in cancer care), 'Physician attitude' (items concerning friendliness, time, personal attention, respect, empathy, attention to the patients loved ones, accuracy, opportunity for the patient to ask questions and trust), 'Accessibility of services' (e.g. access to all professionals involved in various situations), 'Hospital equipment' ('The hospital equipment is modern') and 'Consultation at the emergency room by own doctor'. The largest (18 points) and strongest (ES 0.90) difference between the estimation of health care professionals and patients was found for the single item 'Hospital equipment'. Patients valued modern hospital equipment much higher than health care professionals expected the patients to do.

Patients rated five scales less important than health care professionals expected: 'Support, counseling and rehabilitation' (offering professional support to help patients and their loved ones to deal with emotions and to help patients to re-integrate into their previous daily routine (home, work, school, etc), attention for late effects of treatment)', 'Privacy' (both at the outpatient clinic and on the ward), 'Patient habits', (items concerning individual preferences and requirements, decoration of the room, dietary habits and requirements), 'Patient interest groups' and 'Conveniences' (items concerning the waiting room and the patients room, access to sport and recreation facilities, availability of tea, coffee, soft drinks and soup).

### Comparison of male and female health care professionals

We found gender-related differences within the health care professionals for eight scales and two single items (effect sizes between 0.69 and 1.39) (Table 3). When there were clinically relevant differences, female health care professionals invariably had higher scores. We found the same pattern of gender differences for patients in a previous study [21]. Female patients valued a substantial part of the aspects of care as more important than their male counterparts (Table 3). However, gender differences for patients were much more pronounced and partly for different scales and single items compared to the differences between male and female health care professionals.

**Table 3:** Comparison of the importance scores between female and male patients and female and male health care professionals

	Gender			Gender		
	Patient			Health care professional		
	**Female** **(n = 252)**	**Male** **(n = 134)**	**ES**	**Female** **(n = 47)**	**Male** **(n = 12)**	**ES**

**Scales**						
Mistakes by professionals	92 (13)	87 (14)**	.37	86 (14)	86^1 ^(17)	-
Physician and nurse expertise	90 (10)	88 (11)	-	87 (12)	79 (13)	-
Consultation and transfer	86 (13)	82 (14)*	.30	84 (14)	75 (18)	-
Physician attitude	83 (13)	78 (13)**	.38	77 (12)	71 (16)	-
Patient file confidentiality	84 (18)	75 (19)***	.49	87 (15)	79 (19)	-
Opportunity to choose in care and						
treatment	82 (14)	77 (15)***	.35	78 (14)	77 (8)	-
Nurse attitude	81 (14)	74 (13)***	.51	83 (13)	67 (20)*	1.09
Communication and information	79 (11)	74 (13)***	.43	79 (9)	74 (12)	-
Accessibility of services	78 (14)	73 (13)**	.37	72 (12)	72 (16)	-
Waiting periods	80 (14)	69 (18)***	.71	78 (12)	69 (11)*	.76
Support, counseling and rehabilitation	65 (19)	55 (20)***	.52	69 (13)	58 (17)*	.79
Alternate sources of information	63 (24)	54 (21)***	.39	66 (13)	57 (13)*	.69
Appointments	61 (17)	55 (21)*	.32	64 (13)	57 (16)	-
Rooms and facilities	58 (15)	54 (14)*	.27	58 (12)	41 (13)***	1.39
Food and beverages	56 (19)	56 (19)	-	61 (19)	37 (22)***	1.22
Presence of loved ones	50 (27)	48 (26)	-	58 (22)	50 (17)	-
Privacy	49 (21)	42 (21)**	.33	55 (20)	52 (11)	-
Patient habits	43 (23)	43 (21)	-	54 (17)	37 (20)**	.96
Patient interest groups	40 (23)	32 (22)***	.35	47 (18)	35 (21)	-
Conveniences	37 (17)	36 (16)	-	47 (15)	32 (13)**	1.02
Fellow patient interaction	17 (19)	17 (20)	-	19 (17)	20 (15)	-
**Single items**						
Hospital equipment	83 (20)	84 (20)	-	68 (20)	58 (21)	-
Consultation at ER by own doctor	80 (21)	77 (20)	-	72 (22)	61 (28)	-
Written information	80 (20)	73 (22)**	.34	81 (18)	66 (20)*	.82
Support of a case manager	76 (23)	71 (25)	-	71 (19)	64 (33)	-
Continuity in care	77 (20)	65 (23)***	.57	68 (20)	66 (20)	-
Support by paramedical staff	69 (19)	66 (16)	-	70 (15)	61 (19)	-
Attention for nutrition	68 (22)	67 (22)	-	68 (18)	41 (25)***	1.38
Leaving choices to doctors and nurses	67 (31)	63 (33)	-	61 (29)	63 (26)	-

*p < .05, **p < .01, ***p < .001 (Mann Whitney tests)

ES = Effect Size

- = no statistically significant difference

Generally, female health care professionals did not estimate patients' preferences and needs better than their male colleagues.

## Discussion

Results of this study showed that health care professionals were able to make a correct estimation of the value cancer patients attribute to most aspects of care. In establishing preferences, there was a clear similarity between patients and health care professionals. For both patients and health care professionals safety and the expertise, performance and attitude of doctors and nurses rated highest and the organisational and environmental factors were of relatively less importance. Thus, health care professionals were reasonably well able to make a correct estimation of patients' preferences. From the perspective of delivering patient centered care, these results are certainly encouraging, but there still is room for improvement. We found statistically significant differences for 8 out of 21 scales and 2 out of 8 single items. Health care professionals *underestimated *patients' valuation of the expertise of physicians and nurses, physician attitude, the accessibility of services, a modern hospital equipment and the possibility to be seen by the own doctor directly in case of an emergency. On the other hand, health care professionals *overestimated *the value that patients set on particularly organisational and environmental aspects.

These findings may be of interest to improve care for cancer patients. Our finding suggest that health care professionals may focus too much on aspects of care that patients attach less value to and may pay less attention to aspects that are in the opinion of the patient most important. Failure to tailor care as much as possible to patients' needs and preferences may lead to (unnecessary) dissatisfaction and distress among patients. Patients with unmet needs in the terminal stage of cancer for example, show significantly higher psychological and symptom distress [28].

Prioritization of care aspects by patients is a valid starting point in care renewal processes and may be used to guide decisions in improving care for cancer patients. However, in reorganizing care, the knowledge and experience of health care professionals and logistical and financial constraints should also be taken into account.

Our study also showed that - similar to patients - female health care professionals set higher value on many care aspects than male professionals do. However, in general female health care professionals did not make better estimates of patients' preferences than their male counterparts.

To deliver patient centered care and thereby effectively meet patients needs and wishes, health care professionals should take into account context characteristics of individual patients and customize their services as much as possible. The literature shows that patient characteristics impact upon patients experiences and preferences in health care [21,29-32]. In interpreting the results of this study it is important to be aware of the fact that individual patient characteristics were not taken into account by health care professionals while estimating patient preferences in the questionnaire. The results of the study among patients is an average of 386 respondents and we asked health care professionals to fill out the questionnaire for the average cancer patient. In improving cancer care at the organisational level, health care professionals generally use the average patient as starting point, but standardised care does not meet the need of the individual patient. Further research should therefore focus on the estimation of patient preferences by health care professionals for specific patients or groups of cancer patients (paying attention to the influence of, for example, gender, age, level of education, phase of illness).

There are some limitations of this study. A possible limitation concerning the representativeness of our sample of patients participating in the focus group interviews is the overrepresentation of young patients. It is possible that age may be a confounder in the items addressed. Other potential limitations of this study are the relatively small number of participating health care professionals compared to the number of patients, the (unexplained) low response rate of health care professionals and the relative overrepresentation of nurses and female health care professionals, due to the fact that there are many more nurses working at the departments of medical oncology than physicians and policymakers. Therefore, in future research it is important to expand and confirm these findings in a larger population of health care professionals, including a larger proportion of physicians and policymakers.

## Conclusions

In conclusion, this study showed that health care professionals are reasonably well able to make a correct estimation of cancer patients preferences in general. Nevertheless there are some blind spots. Health care professionals both underestimate and overestimate the value patients attach to a number of care aspects. They should be aware of their own bias and use additional resources to make it possible to optimally differentiate towards patient specific preferences for each patient is different and ultimately the care needs and preferences will also be unique to the person. This indicates the need to develop interventions aimed at supporting professionals in gaining a better insight and understanding of patients' specific preferences, to be fully informed on the patients' preference pattern and thereby to ensure that health care truly meets patients preferences. Furthermore, patients should be encouraged and supported to supply the necessary information for health care professionals to get insight into their specific needs and preferences concerning cancer care.Tailoring care for cancer patients should be a multidisciplinary action of health care professionals and patients to avoid potential biases in perceived needs and preferences of these patients.

## Competing interests

The authors declare that they have no competing interests.

## Authors' contributions

HW: Conception and design, administrative support, provision of study material or patients, collection and assembly of data, data analysis and interpretation and manuscript writing. AdG: Conception and design, provision of study material or patients, data analysis and interpretation and manuscript writing. KW: Conception and design, data analysis and interpretation and manuscript writing. MdH: Administrative support, provision of study material or patients, collection and/or assembly of data, data analysis and interpretation. CK: Data analysis and interpretation and manuscript writing. ST: Provision of study material or patients and manuscript writing. EV: Conception and design, provision of study material or patients, data analysis and interpretation and manuscript writing. All authors read and approved the final manuscript

## Pre-publication history

The pre-publication history for this paper can be accessed here:

http://www.biomedcentral.com/1472-6963/10/198/prepub
